# Prognostic and Functional Analysis of *NPY6R* in Uveal Melanoma Using Bioinformatics

**DOI:** 10.1155/2022/4143447

**Published:** 2022-04-08

**Authors:** ShiMin Mei, Yue Li, Xueran Kang

**Affiliations:** ^1^Department of Ophthalmology, Shanghai Fourth People's Hospital, School of Medicine, Tongji University, Shanghai 200434, China; ^2^Department of General Surgery, Yangpu Hospital, School of Medicine, Tongji University, Shanghai, 200090, China; ^3^Department of Otorhinolaryngology Head and Neck Surgery, Shanghai Ninth People's Hospital, Shanghai Jiao Tong University School of Medicine, Shanghai, China

## Abstract

Neuropeptides can mediate tumor cell proliferation and differentiation through autocrine, paracrine, neurosecretory, and endocrine mechanisms. This study investigated the expression and prognostic significance of neuropeptide Y receptor Y6 (NPY6R) in uveal melanoma (UVM) and preliminarily investigated the biological function of *NPY6R* in UVM. *NPY6R* was poorly expressed in most tumors and was associated with better prognosis in UVM. Among the clinicopathological features of UVM, *NPY6R* expression was lower in male patients. The area under the curve (AUC) value of *NPY6R* for the diagnosis of UVM was 0.676 (95% CI: 0.556–0.795). A nomogram including four clinical predictors was constructed. *NPY6R* expression was significantly associated with features of the UVM immune microenvironment. ESTIMATE and CIBERSORT algorithms were used to calculate the fraction of immune cells and the percentage of infiltration in each patient, respectively. *NPY6R* expression-related gene ontology (GO), Kyoto Encyclopedia of Genes and Genomes (KEGG), and gene set enrichment analyses were performed. GO and KEGG enrichment analyses revealed that *NPY6R*-related genes are mainly enriched in pathways and functions related to visual light perception. Gene set enrichment analysis suggested that *NPY6R* is associated with tumor progression in UVM. *NPY6R* is involved in the tumor progression of UVM and has a good predictive value as a prognostic marker of UVM.

## 1. Introduction

Worldwide, among intraocular malignancies, uveal melanoma (UVM) has the highest prevalence, with an incidence of up to 5.1 per million per year in Caucasians (95–98%) and a lower incidence in Asian and African populations [1–3]. However, in developing countries, UVM incidence has progressively increased recently to that of developed Western countries [4]. UVM has various presentations, many complications, high malignancy, and high invasiveness and metastatic capability, and is associated with a very poor prognosis [5, 6]. UVM metastasis rates are approximately 25% and 34% at 5 and 10 years, respectively, and UVM is associated with an 80% mortality rate within 1 year of metastasis [7–9]. Enucleation, the standard treatment for UVM, is a potentially devastating and disfiguring procedure that confers significant physical and psychological impacts on patients. Nonetheless, the post-enucleation 5-year survival rate of UVM patients is only 17% to 53% [10]. Given the insidious onset and advanced stage at diagnosis, early diagnosis and treatment of UVM are particularly important to improve patient outcomes [11]. Therefore, the identification of molecular markers that mediate the pathogenesis of CM is urgently needed to improve treatment selection and prognosis in UVM patients.

With their wide distribution in the human body, neuropeptides are peptidergic neurotransmitters, modulators, or hormones that modulate the central and peripheral nervous systems [12–15]. As a potential growth factor for normal cells, it can mediate tumor cell proliferation and differentiation through autocrine, paracrine, neurosecretory, and endocrine mechanisms [16, 17]. The role of neuropeptides in tumor development is gaining attention [18]. The *NPY* is susceptible to hypermethylation in certain tumors [19, 20]. Moreover, VIP receptors are overexpressed on the tumor cell surface and promote tumor growth, activat antiapoptotic signaling pathways, and play an important role in regulating the proliferative viability and differentiation of tumor stem cells [21, 22].

In addition, neuropeptides play an important role in ophthalmic diseases [23]. In rat retinal cells, NPY acts in a paracrine manner [24]. whereas, in mice, NPY6R signaling plays a key role in regulating energy homeostasis in the suprachiasmatic nucleus (SCN) [25], which drives circadian rhythms in peripheral organs, including the retina and choroid [26, 27]. Therefore, we hypothesized that the *NPY6R* gene plays an important role in ocular diseases. However, the relevance and biological functions of *NPY6R* in ocular diseases have been rarely reported.

This study investigated the expression and prognostic significance of *NPY6R* in UVM and preliminarily investigated the biological function of *NPY6R* in UVM. We also analyzed the correlation between *NPY6R* expression and the clinical characteristics and prognosis of UVM patients.

## 2. Materials and Methods

### 2.1. Acquisition of Bioinformatic Data

Data on the survival and mRNA expression of the UVM sample from the Cancer Genome Atlas (TCGA) project were obtained. Transcriptome sequencing data and the corresponding clinical information were downloaded from the National Cancer Institute (NCI) Genome Data Portal (GDC Legacy Archive) using the TCGAbiolinks package in R [28], which was used to integrate and normalize the downloaded mRNA expression data to extract information about the *NPY6R* gene. Wilcoxon rank-sum test was used to undertake differential analysis of samples with high and low NPY6R expression and to screen out mRNAs that were differentially expressed in the UVM patients and normal group for enrichment analysis.

### 2.2. Correlation between NPY6R Expression and Clinicopathologic and Survival Analysis

Based on the median NPY6R expression levels, UVM patients were categorized into the NPY6R high expression and NPY6R low expression groups. To investigate the relationship between different clinical characteristics (age, tumor grading, TNM staging, etc.) and NPY6R gene expression and the prognostic risk of UVM patients, Cox univariate and multifactorial regression analyses were performed using the coxph function, and the *p* values and hazard ratio (HR; risk ratio) were calculated. Finally, column line plots for predicting survival were established by the nomogram function in R package based on the results of the Cox multifactor analysis.

### 2.3. Evaluation of Immune Cell Infiltration in Tumor Tissue

The immune microenvironment was scored with reference to previous studies showing [29]. The ESTIMATE algorithm from the R language ESTIMATE package was used to estimate the immune stromal component of the tumor microenvironment estimate (TME) for each sample, which was reported as three scores: ImmuneScore, StromalScore, and ESTIMATEScore. The greater the ratio of the relevant component of the tumor microenvironment, the higher the corresponding score.

Using the ESTIMATE algorithm to determine immune scores for all samples, the deconvolution algorithm based on CIBERSORT (http://http://cibersort.stanford.edu/) calculates the proportion of immune cells infiltrating tumors in each patient in the training and validation sets [30]. Intergroup differences in immune cell infiltration between the high and low NPY6R expression groups in the abovementioned dataset were assessed using the Wilcoxon rank-sum test, and *p* < 0.05 indicated a significant difference in immune cell infiltration. The abovementioned cells were subjected to a follow-up analysis to assess the impact of their infiltration levels on patient prognosis.

### 2.4. Gene Set Enrichment Analysis

The TCGA-UVM expression dataset was loaded into the GSEA JAVA software program, and the number of alignments was set to 1,000 for analysis [31, 32]. The results of gene enrichment at a false discovery rate (FDR) *q* value <25% and nom *p* value <0. 01 were obtained. The JAVA GSEA enrichment analysis software (version 4.0.3) was downloaded from the Broad Institute, and the R language TCGA2STAT package was downloaded from the Comprehensive R Archive Network (CRAN).

### 2.5. GO and KEGG Enrichment Analysis

Based on the risk model, differential genes were obtained for patients in the high- and low-risk groups and were subjected to gene ontology (GO) analysis to obtain differential gene enrichment concerning biological processes, molecular function, and cellular composition. Furthermore, these genes were subjected to Kyoto Encyclopedia of Genes and Genomes (KEGG) enrichment analysis to obtain signaling pathways for differential gene enrichment. The specific methods of enrichment analysis were described in the previous studies [14, 32–35].

### 2.6. Survival and Statistical Analyses

Statistical analysis was performed using R software. Survival curves were plotted based on the NPY6R mRNA expression profile. Survival analysis was performed, and survival curves were plotted using the “survival” package of R language with log-rank test and Cox analysis. The measures are expressed as mean ± standard deviation (*x* ± *s*), and *t* test was applied. The chi-square test was used for statistical data. Wilcoxon rank-sum test was used to compare gene expression levels. Correlations were assessed using Spearman correlation analysis. Factors associated with NPY6R expression were identified using logistic regression analysis as previous researches [36–41]. The predictive power of NPY6R was assessed by receiver operating characteristics (ROC) curves, calibration curves, and clinical decision curves (DCA). *p* < 0.05 was considered indicative of a statistically significant difference.

## 3. Results

### 3.1. Expression Profile of *NPY6R* in Tumors

**Figure 1:**
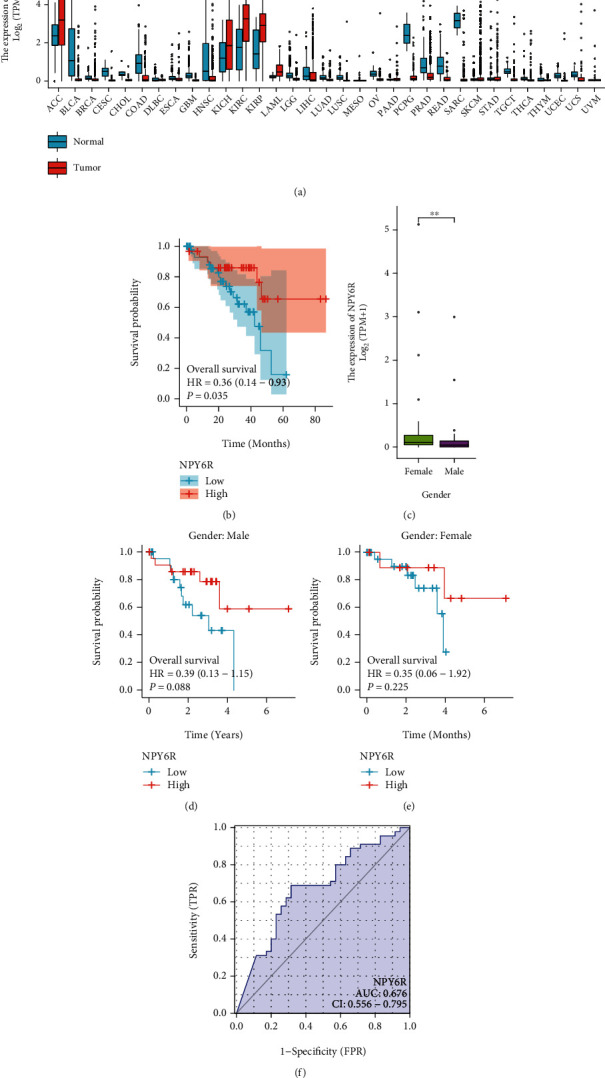
Differential *NPY6R* expression in tumors is associated with poor prognosis in UVM: (a) Expression of *NPY6R* gene in common tumors; (b) *NPY6R* expression levels correlated with the overall survival of UVM patients; (c) levels of *NPY6R* mRNA expression in TCGA database; (d) correlation of *NPY6R* expression levels with overall survival (OS) in female patients with UVM; (e) correlation of *NPY6R* expression levels with OS in male patients with UVM; and (f) ROC curves demonstrating the diagnostic value of NPY6R in UVM.

First, using data from TCGA, we explored the expression of the NPY6R gene in common tumors. NPY6R expression was significantly upregulated in ACC, KICH, KIRC, KIRP, and LAML and significantly downregulated in BLCA, BRCA, CESC, CHOL, COAD, ESCA, GBM, HNSC, LGG, LIHC, LUAD, LUSC, OV, PCPG, PRAD, READ, TGCT, THCA, UCEC, and UCS (Figure 1(a)), which suggests that most tumors have low NPY6R expression. However, there were no control samples of normal tissues in TCGA-UVM dataset. The expression of NPY6R was low in UVM. Kaplan–Meier survival curves showed that patients in the high NPY6R expression group had a higher overall survival rate than patients in the low expression group (Figure 1(b)). Clinicopathological characterization revealed a higher proportion of males among patients with low NPY6R expression and a higher proportion of females among patients with high NPY6R expression (Table 1). In addition, NPY6R expression was nonsignificantly correlated with other factors. Clinical correlation analysis suggested that NPY6R expression was higher in female patients (Figure 1(c)). Logistic regression analysis incorporating NPY6R expression with various pathological characteristics revealed only a sex-linked association for NPY6R (Table 2), which suggests a potentially sex-specific NPY6R expression.

### 3.2. Analysis of the Prognostic Predictive Ability of *NPY6R* and Construction of a Nomogram Prediction Model

**Figure 2:**
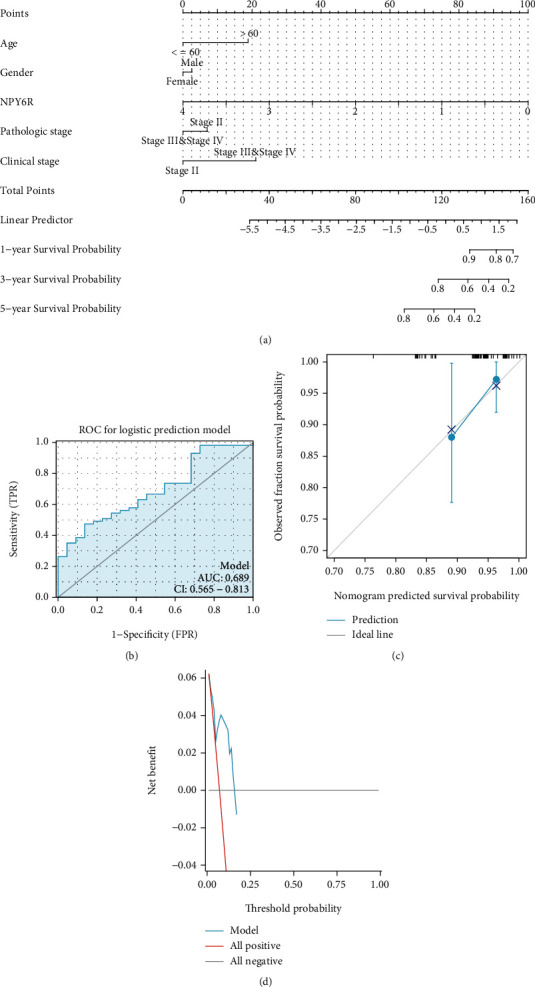
*NPY6R*-based prognostic predictive power analysis and nomogram model construction: (a) a nomogram prediction model for overall survival at 1, 3, and 5 years and (b–d) ROC plots (b), calibration curves(c), clinical decision curves (DCA), and (d) demonstrating the accuracy of this prognostic model in predicting the probability of survival at one year.

As NPY6R expression is sex-specific, subgroup survival analysis suggested that NPY6R was a stronger predictor of overall survival in male patients than in female patients (Figures 1(d) and 1(e)). The ROC curve of *NPY6R* expression and its diagnostic value in UVM is shown in Figure 1(f) (area under the curve [AUC] = 0.676; 95% CI: 0.556–0.795). The nomogram prediction model for overall survival at 1, 3, and 5 years is shown in Figure 2(a) and includes four clinical predictors (age, sex, pathologic stage, and clinical stage). The ROC plot, calibration curve, and clinical decision curve (DCA) demonstrate the accuracy of this prognostic model in predicting the probability of survival at one year (Figures 2(b)–2(d)). The AUC value for this ROC is 0.689 (95% confidence interval: 0.565-0.813) (Figure 2(b)). This suggests that the model has predictive capability. The DCA suggests that the model is helpful in clinical decision making.

### 3.3. Characterization of the Immune Microenvironment of *NPY6R* in UVM

**Figure 3:**
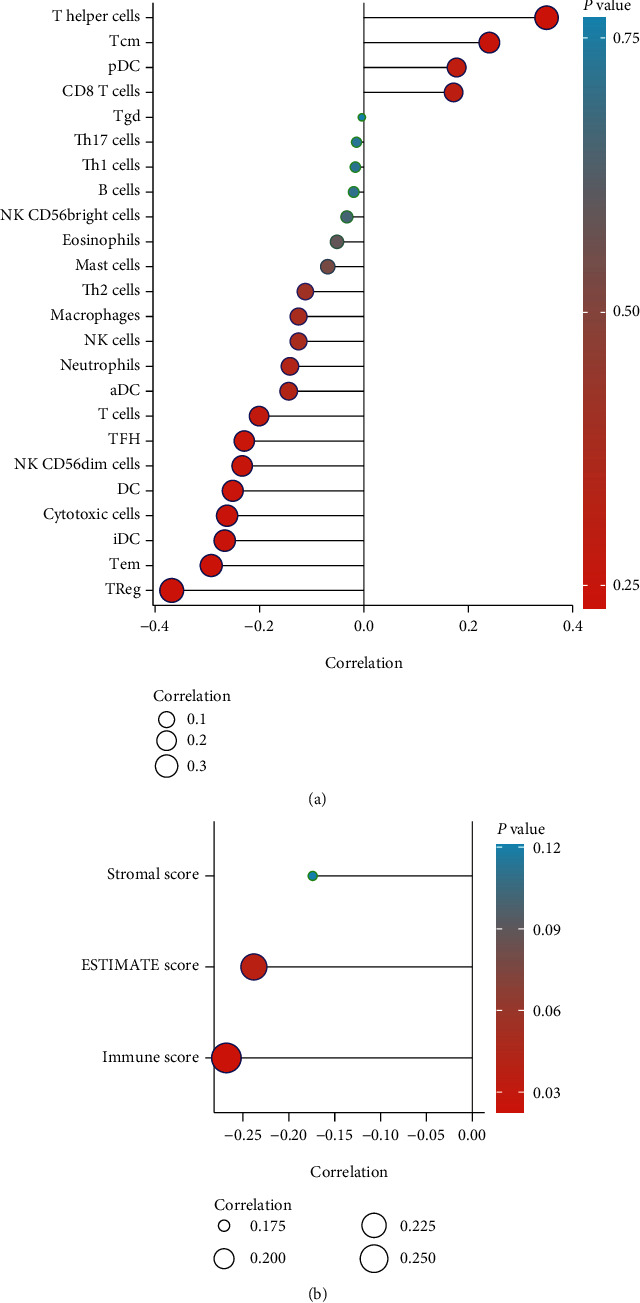
Correlation analysis of *NPY6R* and immune cell infiltration. (a) Correlation analysis of *NPY6R* expression with immune infiltration (colors indicate *p* values and circle sizes indicate correlation coefficients). (b) Correlation analysis of *NPY6R* expression with immune microenvironment scores (StromalScore, ESTIMATEScore, and ImmuneScore).

Correlation analysis between NPY6R expression and tumor immune infiltration suggested that NPY6R expression was significantly and positively correlated with the infiltration of T-helper, Tcm, pDC, and CD8 T cells (Figure 3(a)). (In contrast, NPY6R expression significantly and negatively correlated with the enrichment of T cells, TFH, NK CD56dim cells, DC, cytotoxic cells, iDC, Tem, and TReg (Figure 3(a)). NPY6R expression was significantly and negatively correlated with StromalScore, ESTIMATEScore, and ImmuneScore (Figure 3(b)). NPY6R was found to be significantly and negatively correlated with StromalScore, ESTIMATEScore, and ImmuneScore. This suggests that *NPY6R* expression is significantly associated with the characteristics of the UVM immune microenvironment.

### 3.4. Functional and Pathway Enrichment Analysis Associated with Differential Expression of *NPY6R*

**Figure 4:**
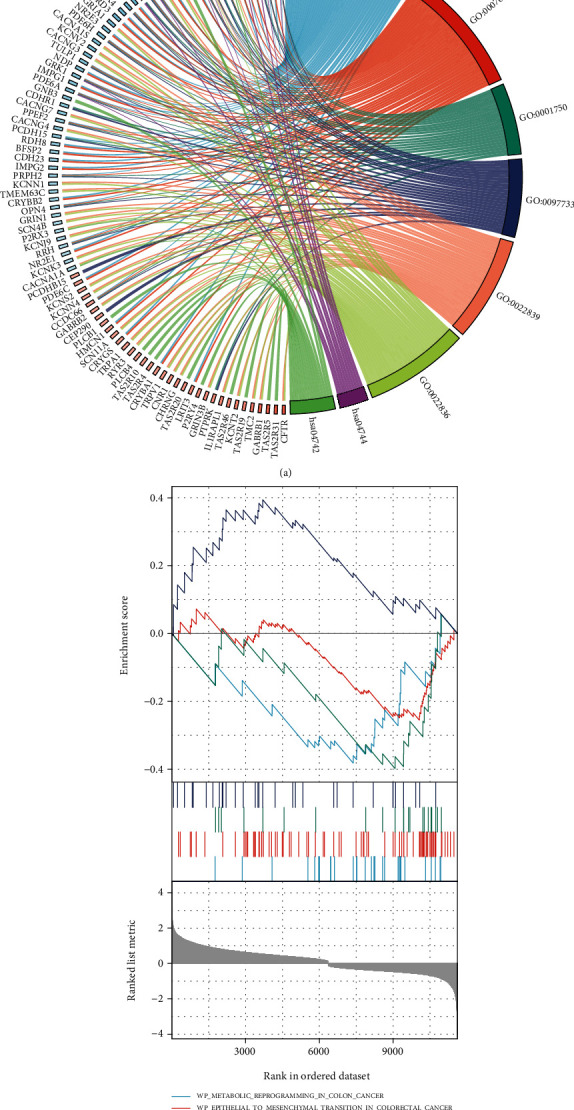
*NPY6R*-related enrichment analysis. (a) Gene ontology (GO) and Kyoto Encyclopedia of Genes and Genomes (KEGG) enrichment analysis of *NPY6R*-associated genes. (b) Enrichment analysis of *NPY6R* expression-related gene set enrichment analysis (GSEA).

To further define the pathway and function of NPY6R enrichment in UVM, genes that are highly correlated with NPY6R expression were obtained by differential analysis. In all, 689 genes met the |log2 (FC)| >1.5 and p.adj<0.05 threshold, with 562 highly expressed (positive logFC) genes and 127 low expressed (negative logFC) genes. GO and KEGG enrichment analysis revealed that the abovementioned genes were mainly enriched in sensory perception of light stimulus (GO:0050953), visual perception (GO: 0007601), photoreceptor outer segment (GO: 0001750), photoreceptor cell cilium (GO: 0097733), ion-gated channel activity (GO: 0022839), gated channel activity (GO:0022836), phototransduction pathway (hsa04744), and taste transduction pathway (hsa04742) (Figure 4(a)), which suggests that these NPY6R-related genes were mainly enriched in pathways and functions related to light visual perception. Furthermore, GSEA was used to analyze the role of NPY6R in UVM. NPY6R expression was associated with metabolic reprogramming in colon cancer (WP), epithelial to mesenchymal transition in colorectal cancer (WP), bladder cancer (KEGG), and constitutive signaling by aberrant PI3K in cancer (REACTOME), which suggests that NPY6R is associated with tumor progression in UVM.

## 4. Discussion

Neuropeptide Y is a bioactive polypeptide consisting of 36 amino acids, with N-terminal proline and C-terminal tyrosine amide, wherein each molecule contains five tyrosine residues, which can maintain a stable conformation in an aqueous solution [42, 43]. Peptides belonging to the neuropeptide Y family can act on multiple G–protein-coupled receptors (e.g., Y1, Y2, Y4, Y5, and Y6), but exert different effects on various receptors [44, 45]. However, the role of *NPY6R* as an NPY receptor needs to be further investigated. In this study, we investigated the expression and function of the *NPY6R* gene in UVM by detecting and analyzing genomic information from public databases.

In this study, low *NPY6R* expression was found in most tumors. In UVM, the overall survival rate of patients in the *NPY6R* high expression group was higher than that of patients in the low expression group. *NPY6R* expression shows a potentially sex-specific association and has some diagnostic and prognostic predictive power in UVM. A nomogram column line graph prediction model that includes four clinical predictive characteristics factors was constructed in this study and showed that *NPY6R* expression was significantly associated with the characteristics of the UVM immune microenvironment. *NPY6R*-related genes are mainly enriched in pathways and functions that are related to visual light perception. GSEA suggested that *NPY6R* expression was associated with tumor progression in UVM.

A previous study revealed no sex difference in UVM [46]. Some studies showed a smaller sex difference in UVM, with a slightly larger number of male than female patients [47–49]. In this study, clinicopathological characterization based on UVM revealed that *NPY6R* expression correlated with sex and was higher in female patients than in male patients, suggesting a potential sex specificity of NPY6R expression. Subgroup survival analysis suggested that the prognostic predictive ability of NPY6R may be more enhanced in male patients with UVM than in female patients.

*NPY6R*, located on chromosome 5q31 and belongs to the cell membrane G–protein-coupled receptor family, is one of the receptors for NPY [50]. Some studies suggested that NPY6R does not have a function in Mansfield [51]. However, *NPY6R* plays an essential role in regulating energy balance and body composition through the VIP-GH/IGF-1 axis [25]. In addition, *NPY6R*, one of the adrenergic candidate sites, may play a role in autonomic/sympathetic dysfunction in the hypertension [52]. In this study, *NPY6R* expression in UVM had a good diagnostic value and was significantly associated with UVM survival prognosis. The overall survival rate of patients with high CM expression was higher than that of patients with low CM expression. Previous studies revealed that metastasis was a major cause of death in CM patients [9, 53]. G–protein-coupled receptors are an important factor in tumor growth and are metastasized [54]. NPY6R, which belongs to the G–protein-coupled receptor family, may affect the survival prognosis of patients through its involvement in the development of UVM, although the exact mechanism needs to be further investigated.

Current clinical treatments for UVM include chemotherapy, radiotherapy, immunotherapy, and targeted molecular therapy. Due to the dual protection of low mutational load and “immune immunity,” UVM immunotherapy is not very effective [55]. A study found that mUM patients treated with anti-PD-1 monoclonal antibodies comprised 3.6% partial responders and 8.9% participants with stable disease, with median progression-free survival and overall survival of 2.8 and 7.6 months, respectively, which indicated that the prognosis of immunotherapy patients did not improve significantly [56]. However, the results of the first prospective study of the therapeutic value of ipilimumab in patients with Class 2 genotype UVM that was conducted by Fountain et al. revealed that immunotherapy reduced the risk of distant metastases in high-risk lesions [57]. Furthermore, tumor vaccines for the treatment of UVM are currently being investigated. The dendritic cell-containing vaccine was administered to patients with primary UVM combined with trisomic monosomy, and most of the patients developed tumor-specific immune responses without serious adverse effects, and progression-free survival and overall survival were significantly prolonged, confirming that dendritic cell immunization is safe and effective in patients with high-risk UVM [58]. In addition, Chandran et al. found that regression of metastatic UVM tumors could be induced by transplantation of autologous tumor-infiltrating lymphocytes (TILs) [59]. Similar to these studies, in our study, NPY6R expression significantly correlated with the characteristics of the UVM immune microenvironment and could be a new target for UVM immunotherapy.

With the rapid development of molecular biology, the molecular genetic study of UVM has progressed, although the mechanisms of action, regulation, and metastasis have not been fully elucidated. PI3K expression is significantly increased in choroidal melanoma tissues and is closely related to the invasion and migration of cancer cells [60]. In animal models of choroidal melanoma, inhibition of the PI3K/MMP signaling pathway significantly reduced tumor cell growth and neovascularization [61]. Yan et al. found that high expression of insulin-like growth factor-1 (IGF-1) in patients with metastatic UVM upregulated the phosphorylation levels of Akt, mTOR, and PI3K, which are key factors of the PI3K/Akt signaling pathway [62]. In contrast, PI3K-related signaling pathways play an important role in retinal diseases. As a downstream target of PI3K, Akt kinase plays a key role in the development and protection/regeneration of retinal ganglion cells [63]. Furthermore, the PI3K/Akt signaling pathway is involved in the pathology of retinal detachment [64]. Therefore, we speculate that retina-related pathways may play a role in UVM progression, as supported by the results of the present study.

In summary, this study provides the first multilevel, multifaceted analysis of the role of NPY6R in UVM through information mining of databases. NPY6R was associated with UVM prognosis, and a novel UVM prognosis prediction nomogram prediction model was constructed. NPY6R was significantly associated with the characteristics of the UVM immune microenvironment and was involved in UVM tumor progression. However, some limitations of this study need to be acknowledged: (i) the study was analyzed using bioinformatics, and the specific mechanism underlying the correlation between NPY6R and prognosis and mortality should be investigated in animal experiments; (ii) clinical specimens were not collected in this study to verify the results obtained using bioinformatics; and (iii) the accuracy of the NPY6R-based model prediction model needs to be further optimized.

## 5. Conclusion

In this study, we used bioinformatics to initially confirm the correlation between NPY6R and UVM prognosis, construct a nomogram line graph prediction model, and analyze the possible mechanisms and NPY6R-related pathways involved in the progression of UVM. Therefore, this study provides new insights for the use of NPY6R in the early diagnosis, treatment, and prognosis prediction of UVM.

## Figures and Tables

**Table 1:** Relationship between the expression of NPY6R and clinicopathological features.

Characteristic	Low expression of NPY6R	High expression of NPY6R	*p*
*n*	40	40	
Age, mean ± SD	61.2 ± 12.73	62.1 ± 15.22	0.775
Pathologic T stage, *n* (%)			0.398
T2	6 (7.5%)	8 (10%)	
T3	14 (17.5%)	18 (22.5%)	
T4	20 (25%)	14 (17.5%)	
Pathologic N stage, *n* (%)			1.000
N0	26 (32.9%)	26 (32.9%)	
NX	14 (17.7%)	13 (16.5%)	
Pathologic M stage, *n* (%)			0.591
M0	26 (33.3%)	25 (32.1%)	
M1	1 (1.3%)	3 (3.8%)	
MX	13 (16.7%)	10 (12.8%)	
Pathologic stage, *n* (%)			0.331
Stage II	18 (22.8%)	21 (26.6%)	
Stage III	21 (26.6%)	15 (19%)	
Stage IV	1 (1.3%)	3 (3.8%)	
Clinical T stage, *n* (%)			0.262
T2	3 (3.8%)	1 (1.3%)	
T3	15 (19.2%)	21 (26.9%)	
T4	22 (28.2%)	16 (20.5%)	
Clinical N stage, *n* (%)			0.615
N0	37 (46.2%)	39 (48.8%)	
NX	3 (3.8%)	1 (1.2%)	
Clinical M stage, *n* (%)			1.000
M0	37 (46.2%)	36 (45%)	
M1	1 (1.2%)	2 (2.5%)	
MX	2 (2.5%)	2 (2.5%)	
Clinical stage, *n* (%)			0.535
Stage II	17 (21.2%)	19 (23.8%)	
Stage III	22 (27.5%)	18 (22.5%)	
Stage IV	1 (1.2%)	3 (3.8%)	
Gender, *n* (%)			0.007
Female	11 (13.8%)	24 (30%)	
Male	29 (36.2%)	16 (20%)	
Weight, *n* (%)			0.218
≤80	10 (18.9%)	17 (32.1%)	
>80	15 (28.3%)	11 (20.8%)	
Height, *n* (%)			0.996
≤170	16 (30.2%)	19 (35.8%)	
>170	9 (17%)	9 (17%)	
BMI, *n* (%)			1.000
≤30	18 (34%)	21 (39.6%)	
>30	7 (13.2%)	7 (13.2%)	
Histological type, *n* (%)			0.949
Epithelioid cell	6 (7.5%)	7 (8.8%)	
Spindle cell	15 (18.8%)	15 (18.8%)	
Mix	19 (23.8%)	18 (22.5%)	
Tumor shape, *n* (%)			0.408
Diffuse	0 (0%)	2 (3.9%)	
Dome	20 (39.2%)	16 (31.4%)	
Mushroom	6 (11.8%)	7 (13.7%)	
OS event, *n* (%)			0.621
Alive	27 (33.8%)	30 (37.5%)	
Dead	13 (16.2%)	10 (12.5%)	
DSS event, *n* (%)			1.000
Alive	29 (36.2%)	30 (37.5%)	
Dead	11 (13.8%)	10 (12.5%)	
PFI event, *n* (%)			0.817
Alive	26 (32.5%)	24 (30%)	
Dead	14 (17.5%)	16 (20%)	

**Table 2:** Logistic regression analysis of the expression of NPY6R and various pathological features.

Characteristics	Total(N)	Odds ratio(OR)	*p* value
Pathologic T stage (T4 vs. T2 and T3)	80	0.538 (0.216-1.312)	0.177
Pathologic N stage (NX vs. N0)	79	1.163 (0.458-2.975)	0.750
Pathologic M stage (M1 and MX vs. M0)	78	1.212 (0.475-3.110)	0.687
Pathologic stage (stage III and stage IV vs. stage II)	79	0.701 (0.287-1.697)	0.432
Clinical T stage (T4 vs. T2 and T3)	78	0.595 (0.240-1.450)	0.256
Clinical M stage (M1 vs. M0)	76	2.056 (0.189-45.378)	0.563
Clinical stage (stage III and stage IV vs. stage II)	80	0.817 (0.336-1.974)	0.653
Gender (male vs. female)	80	0.253 (0.096-0.633)	0.004
Age (>60 vs. ≤60)	80	1.494 (0.621-3.641)	0.372
BMI (>30 vs. ≤30)	53	0.857 (0.248-2.954)	0.805
Tumor shape (dome vs. diffuse and mushroom)	51	0.533 (0.150-1.791)	0.314

## Data Availability

UVM sample survival and mRNA expression data were included in The Cancer Genome Atlas (TCGA) project. Transcriptome sequencing data and clinical information were downloaded from the National Cancer Institute (NCI) Genome Data Portal (GDC Legacy Archive).

## Abbreviations

GO: Gene oncology
GPCR: G protein-coupled receptors
KEGG: Kyoto encyclopedia of genes and genomes
NPY6R: Neuropeptide Y receptor Y6
OS: Overall survival
PFS: Progression-free survival
SCN: Suprachiasmatic nucleus
TCGA: The cancer genome atlas
UVM: Uveal melanoma
ROC: Area under the curve.
